# Hybrid Genome Sequencing and Comparative Analysis of Three Novel *Listeria monocytogenes* Strains: Insights into Lineage Diversity, Virulence, Antibiotic Resistance, and Defense Systems

**DOI:** 10.3390/foods15010088

**Published:** 2025-12-28

**Authors:** Violeta Pemaj, Aleksandra Slavko, Konstantinos Konandreas, Dimitrios E. Pavlidis, Anastasios Ioannidis, Konstantinos Panousopoulos, Nikoletta Xydia, Vassiliki Antonopoulou, Marina Papadelli, Eleftherios H. Drosinos, Panagiotis N. Skandamis, Simon Magin, Konstantinos Papadimitriou

**Affiliations:** 1 Laboratory of Food Quality Control and Hygiene, Department of Food Science and Human Nutrition, Agricultural University of Athens, Iera Odos 75, 11855 Athens, Greece; v.pemaj@go.uop.gr (V.P.); dockonand@gmail.com (K.K.); ehd@aua.gr (E.H.D.); pskan@aua.gr (P.N.S.); 2 Department of Food Science and Technology, University of the Peloponnese, 24100 Antikalamos, Greece; a.slavko@go.uop.gr (A.S.); d.pavlidis@go.uop.gr (D.E.P.); vinopanuk@gmail.com (K.P.); m.papadelli@go.uop.gr (M.P.); 3 Laboratory of Clinical Microbiology, School of Medicine, Attikon University Hospital, National and Kapodistrian University of Athens, Rimini 1, 12462 Chaidari, Greece; tasobi@med.uoa.gr; 4 2nd Indernal Medicine Clinic, General Hospital of Kalamata, 24100 Antikalamos, Greece; nikolxds@gmail.com; 5 Microbiology Department, General Hospital of Kalamata, 24100 Antikalamos, Greece; vassoanto@gmail.com; 6 Institute for Artificial Intelligence in Medicine (IKIM), University Hospital Essen, University of Duisburg-Essen, 45131 Essen, Germany; simon.magin@uk-essen.de

**Keywords:** Comparative genomics, *L. monocytogenes*, genetic diversity, bioinformatics, virulence factors, antimicrobial resistance

## Abstract

*Listeria monocytogenes* is a major foodborne pathogen, responsible for severe listeriosis outbreaks associated with contaminated foods. This study reports the comparative genomic analysis of three novel *L. monocytogenes* strains C5, A2D9 and A2D10, obtained from dairy and clinical sources. Hybrid genome sequencing with Oxford-Nanopore and Illumina technologies provided high-quality complete chromosomes. Phylogenomic analysis revealed a highly conserved core genome alongside accessory genome diversity. Strain C5 belonged to sequence type ST2, while A2D9 and A2D10 were assigned to ST155 and ST1, respectively. All strains exhibited close genomic relatedness to isolates from dairy animals and/or the dairy environment. Functional analysis identified conserved metabolic functions across all genomes. A total of 40 virulence genes were detected, including the LIPI-1 island in all strains and the LIPI-3 operon exclusively in A2D10, indicating a potential hypervirulent phenotype consistent with its ST1 background and the associated fatal clinical outcome. All strains exhibited similar antimicrobial resistance profiles typical of *L. monocytogenes* and diverse defense systems. The newly sequenced strains provide a valuable resource for functional analyses of the mechanisms underlying adaptation of *L. monocytogenes* to diverse environments.

## 1. Introduction

*Listeria monocytogenes* is a Gram-positive, facultative intracellular pathogen that causes listeriosis, a severe foodborne disease with high mortality rates among vulnerable populations such as neonates, pregnant women, elderly, and immunocompromised individuals [1,2,3]. This bacterium thrives in different environments, and it is primarily transmitted to humans through the consumption of contaminated foods, such as soft cheeses, smoked fish, and ready-to-eat (RTE) products. It has the ability to survive in a variety of food environments due to its ability to tolerate cold, high salinity, and disinfectants [1,4]. While cases of listeriosis remain relatively rare, it is of major public health concern due to the severity of clinical outcomes and the challenges associated with its control in the food chain [1].

The genomic architecture of *L. monocytogenes* reflects its ecological adaptability and pathogenic versatility. Its genome consists of a circular chromosome approximately of 2.9 Mb in size, with a high coding density and relatively conserved gene order across strains [5]. However, extensive comparative genomic analyses have revealed substantial interstrain variability, largely attributed to mobile genetic elements such as prophages, genomic islands, and plasmids. These elements contribute to genome plasticity, influencing traits such as stress tolerance, antimicrobial resistance, and virulence [5].

*L. monocytogenes* strains are classified into four major evolutionary lineages (I–IV), four molecular serogroups (IIa, IIb, IIc and IVb) and 12 serotypes (1/2a, 1/2b, 1/2c, 3a, 3b, 3c, 4a, 4b, 4c, 4d, 4e and 7), which are associated with distinct, but overlapping ecological niches and clinical outcomes [6,7,8]. Lineage I includes strains with the highest virulence potential [1]. Most strains belong to lineages I and II. Serotypes 1/2b and 4b (lineage I) and 1/2a (lineage II) are frequently associated with human listeriosis cases [6,8]. Lineage II strains, particularly serotype 1/2a, are also commonly isolated from food and farm environments and are responsible for cases of listeriosis in both humans and animals [8,9]. In contrast, lineages III and IV are predominantly associated with animal hosts and are not common [7,8,10].

Comparative studies have demonstrated lineage-specific differences in genome content, including the presence or absence of key virulence factors, metabolic genes, and regulatory systems. For example, the master regulator *prfA*, listeriolysin O (*hly*) and internalin genes (*inlA*, *inlB*) are central to host invasion and intracellular survival, but their sequence and expression can vary significantly between strains, influencing pathogenic potential [11,12]. *L. monocytogenes* also exhibits an intrinsic antimicrobial resistance (AMR) background characterized by the presence of core genes such as *fosX*, *norB*, *mprF*, *lin*, and *mdrL*, suggesting resistance to multiple antibiotic classes [13,14]. *L. monocytogenes* can harbor different classical antiviral defense systems like restriction-modification (R-M) and CRISPR-Cas [5,15,16,17]. Multiple defense systems can also be combined in a strain-specific manner [18].

Despite the availability of many sequenced genomes, questions remain regarding the evolutionary forces shaping genome reduction, the role of horizontal gene transfer, and the extent of genomic conservation across environmental and clinical isolates. While some *L. monocytogenes* strains exhibit streamlined genomes with loss of accessory genes, others maintain large, mosaic structures enriched with horizontally acquired elements. Understanding these differences is essential to unravel the molecular mechanisms underlying adaptation and virulence [19].

In the present study, we conducted a comparative genomic analysis of three novel *L. monocytogenes* strains, comprising one isolate obtained from a dairy farm environment and two clinical isolates most likely originating from foodborne infections. Our objective was to generate high-quality, complete genome assemblies with minimal gaps and assembly errors using a hybrid sequencing approach that combined Oxford Nanopore Technologies (ONT) long reads with Illumina short reads. We also aimed to investigate in depth key genomic features, including the distribution of virulence-associated genes, antimicrobial resistance determinants, and other adaptive traits. These newly characterized strains expand the genomic landscape of *L. monocytogenes* and may support future comparative and functional studies.

## 2. Materials and Methods

### 2.1. Isolation and Antibiotic Resistance Testing

A total of three *L. monocytogenes* strains were used in this study. Specifically, *L. monocytogenes* C5 was previously isolated from the dairy farm environment as already described [20], while two additional clinical strains A2D9 and A2D10 were isolated from patients with listeriosis, most likely of foodborne origin (please see below). Strain A2D10 was associated with a fatal clinical outcome.

Strains were cultivated in Brain Heart Infusion (BHI) medium (Oxoid, Basingstoke, UK) at 37 ± 0.2 °C overnight, and genomic DNA was extracted with the NucleoSpin DNA RapidLyse kit (Macherey-Nagel, Düren, Germany). DNA integrity and approximate fragment size were assessed on agarose gels. Their antimicrobial susceptibility testing was assessed based on the European Committee on Antimicrobial Susceptibility Testing (EUCAST) using the standardized disk diffusion method [21].

### 2.2. Complete Genome Sequencing

The complete genomes of *L. monocytogenes* strains were obtained through a hybrid assembly approach using both ONT long-read and Illumina short-read sequencing technologies.

Long-read sequencing was performed on a GridION X5 Mk1 device (ONT, Oxford, UK) using R9.4.1 flow cells and chemistry (FLO-MIN106, SQK-LSK109, EXP-NBD104, EXP-NBD114). DNA isolates were measured by fluorometry (Qubit4, Thermo Fisher Scientific, Waltham, MA, USA) and spectrophotometry (NanoDrop One, Thermo Fisher) to obtain precise quantification and purity data. 600 ng of each bacterial sample was used as initial input for the library preparation workflow. Nanopore sequencing preparation followed standard ONT protocols including multiple cleanups. Appropriate bead ratios and long fragment buffer were employed to enrich for longer fragments. In brief, fragments were first end-prepped and then barcoded. Barcoded samples were pooled using equal DNA mass (100 ng) and ligated to sequencing adapters. Approximately 50 fmol of final library, based on fragment length estimation, was loaded onto the flow cell. Strains were sequenced to 166× (A2D10), 221× (A2D9) and 251× (C5) coverage with Nanopore, allowing for selection of the longest, highest quality reads for assembly.

Short-read sequencing was conducted by Novogene (Cambridge, UK) using the Illumina NovaSeq 6000 platform (Illumina, San Diego, CA, USA) with 2 × 150 bp paired-end reads, yielding greater than 300x coverage for all bacterial genomes. Library preparation followed standard workflows including adapter ligation, sequencing and quality control.

### 2.3. Bioinformatics Analysis

A dedicated snakemake [22] workflow (https://github.com/simakro/CobraHy) was implemented for efficient and reproducible data processing of each sample. The workflow takes long Nanopore and short Illumina reads as input to generate high quality hybrid assemblies and includes taxonomic classification, plasmid identification, genomic annotation and quality assessment. In detail Nanopore long reads were subjected to quality control and adapter trimming with NanoPlot [23] and Porechop [24], respectively. Illumina reads also underwent adapter trimming and quality control with FastQC [25]. Long reads were assembled using Flye [26], to generate the initial draft assembly, which was subsequently polished first with nanopore data using Medaka [27], then with Illumina short reads aligned with the bwa-mem2 2.2.1 software package [28], in three iterations of Pilon [29]. All contigs were checked for circularity and oriented using the Circlator tool [30]. Plasmid identification was performed with PlasClass [31]. Assemblies were taxonomically classified with GTDBtk [32], and the closest reference genome was automatically retrieved. The reference was used to guide annotation with Prokka [33], as well as assessment of completeness with BUSCO [34]. Two assemblies were generated for each strain using the described snakemake workflow.

To maximize contiguity and precision of the primary assembly, an optimized read selection algorithm was employed to choose nanopore reads with the highest length and quality until a coverage of 60x was reached, yielding minimum read lengths and qualities of 15 kb and Q15 (A2D9 and C5), and 12 kb and Q12 (A2D10), respectively. A secondary assembly, employing a lower size cutoff of 700 bp, was performed to facilitate detection of smaller plasmids potentially missed in the primary assembly due to selection for very long reads.

The generated primary assemblies and associated output of the snakemake workflow were used for further and in-depth analysis. Completeness and contamination of assemblies were assessed with CheckM2 v 1.1.0. with the UniRef100 v1. reference database [35]. Clustering of strains was conducted with the Type (Strain) Genome Server (TYGS) [36], including the *L. monocytogenes* EGD-e as reference strain (accession number NZ_CP023861.1). The genomes of strains were aligned and visualized using progressiveMauve [37] and Circoletto [38] with default BLAST parameters. Comparative and functional genomics were performed using EDGAR 3.5 to compute the pan-genome, core-genome and average nucleotide identity (ANI) [39,40]. Typing of strains was performed using the BIGSdb-Lm [2]. JSpeciesWS was used to identify the closest related genomes to each of the three *L. monocytogenes* strains [41] with tetra correlation search (TCS). Additionally, multilocus sequence typing (MLST) and core-genome multilocus sequence typing (cgMLST) were carried out on assembled genomes using the CLC genomics workbench v24.0.2 (Qiagen, Hilden, Germany), employing *L. monocytogenes* MLST [42] and cgMLST schemes. Reconstruction of metabolic pathways was achieved through BlastKOALA using the KEGG v116.0 database [43]. Clusters of orthologous groups (COG) functional categories were obtained with eggNOG-mapper v2 [44] against the eggNOG 5.0 database. Finally, genes related to virulence and antimicrobial resistance (AMR) were also identified with ABRicate v1.0.1 [45] against the ARG-ANNOT [46], CARD [47], MEGARes [48], and VFDB databases [49]. Only genes meeting the default cut-off criteria of ABRicate (sequence identity ≥80% and alignment coverage ≥80%) were considered present in the genome. Defense mechanisms were explored with the DefenseFinder [50], prophage regions using PHASTER tool [51] and genomic islands (GI) were predicted by IslandViewer 4 [52]. All tools were run with default parameters unless otherwise specified.

## 3. Results and Discussion

### 3.1. General Characteristics of the Novel L. monocytogenes Complete Genomes

According to CheckM2, all three assemblies displayed excellent completeness (>99.9%) and minimal contamination (<1%) (Table 1) with a GC content of 38%, which is consistent with previous reports for *L. monocytogenes* [53].

Further genome annotation using Prokka revealed highly consistent genomic features across the three *L. monocytogenes* strains (Table 2).

The strains contained between 2837 and 2971 predicted genes, of which 2769 to 2903 were identified as protein-coding sequences (CDS). In all strains, 67 tRNA genes and 18 rRNA genes were identified, indicating a conserved core genomic organization of RNA-coding genes. These results are in accordance with the previous studies of *L. monocytogenes* genomes [5]. Finally, no plasmids were identified in any of the strains.

### 3.2. Phylogenomic and Whole-Genome Comparative Analysis

**Figure 1:**
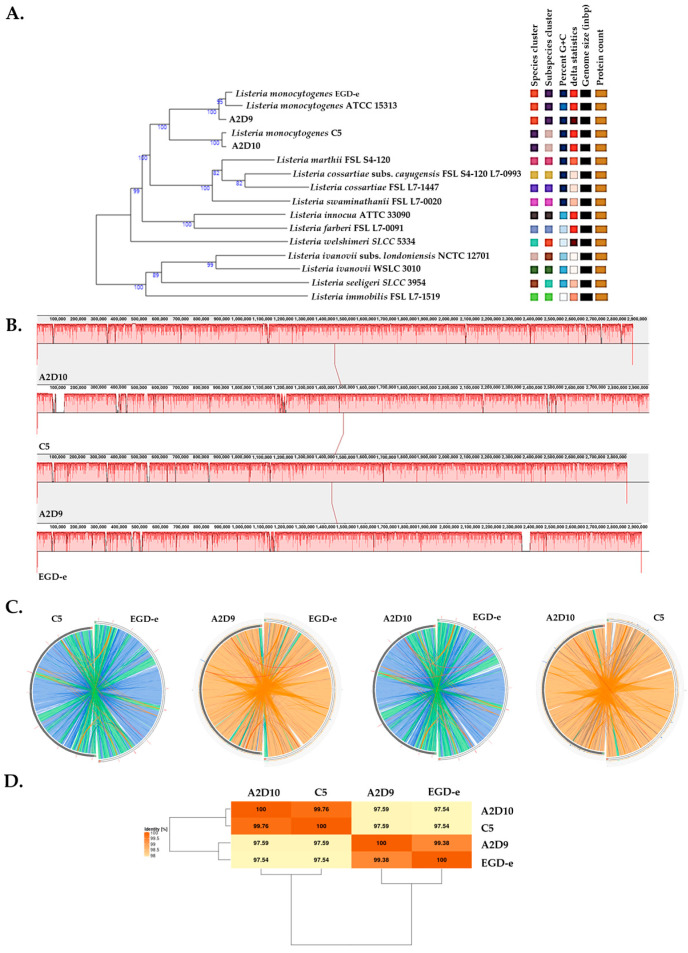
Phylogenomic and whole-genome comparative analysis of *L. monocytogenes* strains C5, A2D9, A2D10 and the reference strain EGD-e. (**A**) Phylogenetic tree among the *L. monocytogenes* strains according to TYGS database. (**B**) Mauve genome alignment of strains. (**C**) Circular genome alignments between strains. Colored ribbons indicate different levels of nucleotide identity based on the BLASTn local alignments (blue: ≤95%, green: ≤98%, orange: ≤99.99% and red: >99.99%). (**D**) ANI matrix of *L. monocytogenes* strains.

Phylogenomic analysis of *L. monocytogenes* revealed the evolutionary relationships among the *L. monocytogenes* strains and the type strains from the TYGS database (Figure 1A). Genome alignments with Mauve suggested a uniform coverage across most genomic regions (Figure 1B). To complement these observations, Circoletto was employed to visualize pairwise genomic alignments between the reference and each of the other strains (Figure 1C). This analysis clearly indicated the close relatedness of EGD-e and A2D9, as well as C5 and A2D10. The ANI matrix results indicated that all pairwise comparisons exceeded 98%, confirming the pairs of strains showing closer relations as suggested by the Circoletto analysis (Figure 1D).

### 3.3. Typing of L. monocytogenes Strains

Initial typing with BIGSdb-Lm predicted the classification of strains in two serogroups. Strains C5 and A2D10 were most closely related to serogroup IVb, while A2D9 to serogroup IIa. *L. monocytogenes* strains belonging to serogroups IVb and IIa have been previously isolated from ready-to-eat foods of animal origin, raw meat and food production environments [6,54].

Further investigation using JSpeciesWS revealed that genomes of each of *L. monocytogenes* strains showed high similarity (Z-score ≥ 0.999) to *L. monocytogenes* originating from specific environments. Strain C5 displayed the strongest correlation with *L. monocytogenes* Scott A isolated from milk during a listeriosis outbreak [55] and ATCC 19117 isolated from sheep [56]. Strain A2D9 showed the closest genomic relationship to foodborne isolates *L. monocytogenes* Finland 1998 [57] and QOC2 [58], both linked to outbreaks associated with the consumption of dairy products. Finally, strain A2D10 showed the highest similarity to *L. monocytogenes* strains NTSN and F2365, which were originally isolated from sheep and cheese, respectively [59,60]. The close genomic relationship between the studied strains and those associated with the dairy environment suggests that they may have originated from this environment as well.

**Figure 2:**
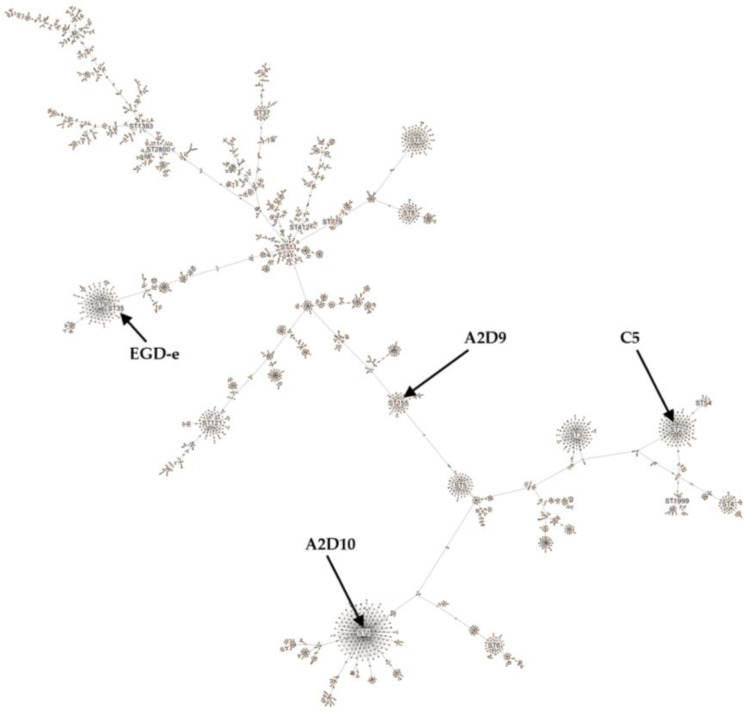
Minimum spanning tree constructed according to *L. monocytogenes* MLST scheme.

The strains were further compared using the MLST *L. monocytogenes* scheme with seven housekeeping genes (*abcZ*, *bglA*, *cat*, *dapE*, *dat*, *ldh*, and *lhkA*) [61] (Figure 2). *L. monocytogenes* C5 strain was assigned to sequence type (ST) 2 of clonal complex (CC) 2 [62]. Isolates belonging to ST2 have been detected in dairy, meat, and fish products [63,64,65], and clinical cases [66]. Strain A2D9 was assigned to ST155 of CC155 [61], which has been identified in fish, meat products and clinical samples [67,68]. *L. monocytogenes* A2D10 strain was assigned to ST1 of hypervirulent CC1 [59,65,69,70]. ST1 strains have been verified to be associated with clinical cases [61], as well as with food-related environments [71,72]. *L. monocytogenes* EGD-e was assigned to ST35 of CC9 as previously described [73]. Members of ST35 have been associated with food products of animal origin [69,74]. Finally, cgMLST analysis assigned *L. monocytogenes* C5 to cgST9209, A2D9 to cgST21252, and A2D10 to cgST32884, although these assignments were inconclusive. The inconclusiveness arises from the presence of several nearly identical cgMLST profiles in the database, each exhibiting minimal allelic differences and comparable k-mer similarity scores. As more *L. monocytogenes* genomes are deposited and assigned to cgMLST profiles in future updates, the increased database coverage may help refine these assignments.

The comparative genomic and MLST analyses revealed that all three newly sequenced *L. monocytogenes* strains share close genomic relationships with isolates previously recovered from dairy and other food-related environments. Although strains A2D9 and A2D10 were obtained from clinical cases, their clustering with foodborne and dairy-associated reference strains suggests a possible food-related origin or transmission pathway, consistent with the well-documented ability of *L. monocytogenes* to cross the food chain barrier and cause human infection.

### 3.4. Pan-Genome and Core-Genome Analysis

**Figure 3:**
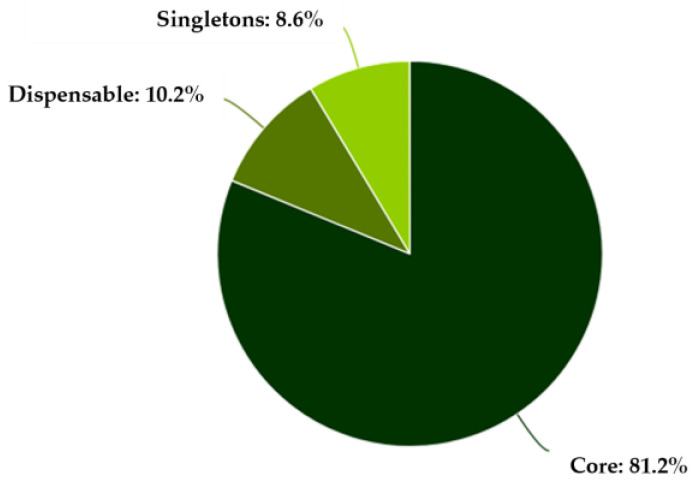
Pan-genome distribution among the *L. monocytogenes* strains.

Comparative genomic analysis using EDGAR 3.5 was performed on the three strains and the reference genome. The results showed that the genomes were highly conserved, sharing a large proportion of common genes in the core-genome, while only a small fraction of genes were unique to individual isolates (the accessory genome) (Figure 3). The analysis revealed that the core-genome accounted for approximately 80% of all genes, indicating a very close genetic relationship between the isolates.

**Figure 4:**
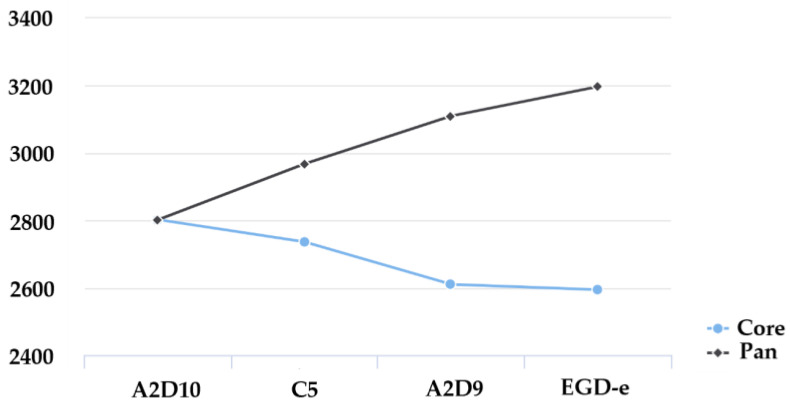
Comparison between the core-genome (blue line) and the pan-genome (black line) among the *L. monocytogenes* strains.

Additionally, the comparison between the core-genome remained relatively stable showing a slight decline with each added genome (Figure 4). Pan-genome continued to increase with the addition of more strains. These results indicate considerable genomic diversity among the isolates, supporting the concept of *L. monocytogenes* as a species with an evolving and expanding gene repertoire.

**Figure 5:**
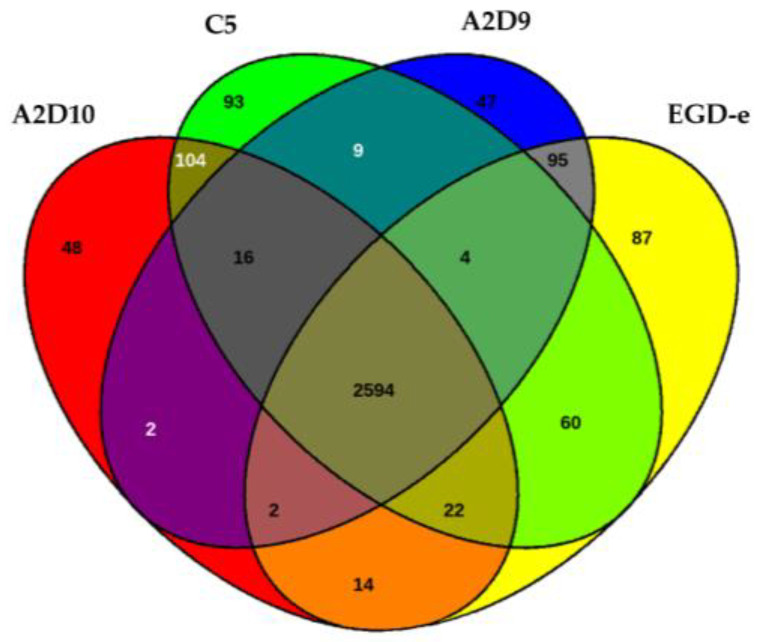
Venn diagram among all the genes of *L. monocytogenes* strains.

The Venn diagram displaying the distribution of orthologous genes indicated that 2594 genes were shared by all strains (Figure 5).

Surrounding regions denoted strain-specific or partially shared genes. For instance, strain A2D10 possessed 48 unique genes and A2D9 had 47, while *L. monocytogenes* C5 harbored 93 genes absent from the other isolates. The reference genome also contributed 87 unique coding sequences. Intermediate overlaps, reflected genes conserved in subsets of the strains, indicating functional divergence and variable genome content.

### 3.5. Functional Annotation

**Figure 6:**
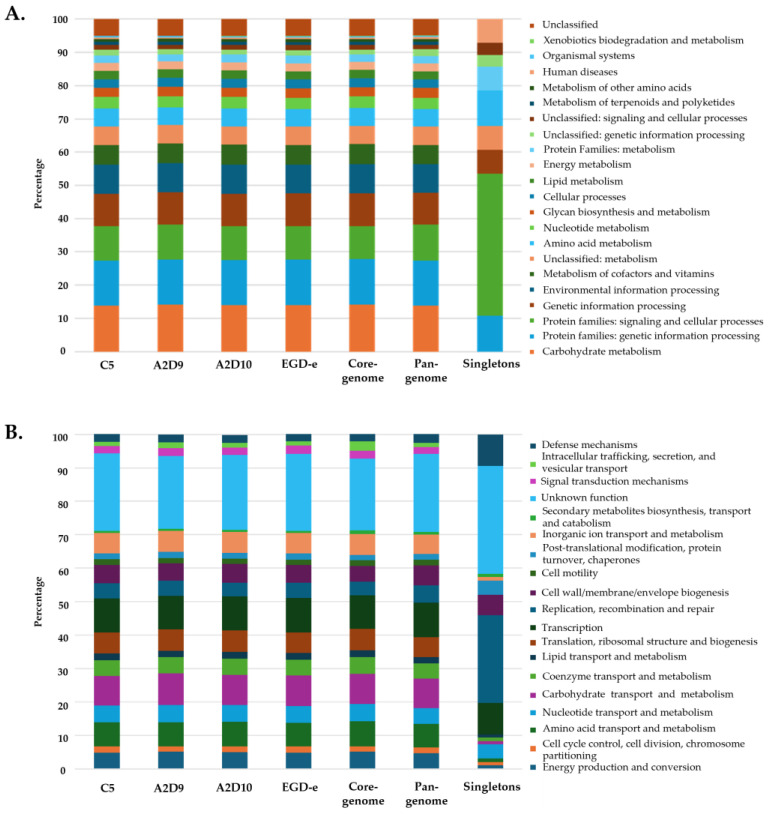
Functional annotation of *L. monocytogenes* strains based on (**A**) KEGG pathway categories and (**B**) COG functional categories.

Functional analysis revealed that proteins from all three *L. monocytogenes* strains and the reference strain were assigned to similar KEGG pathways. Most genes were found related to metabolic functions, including carbohydrate, amino acid, energy, nucleotide and lipid metabolism, as well as genetic and environmental information processing and cellular processes (Figure 6A). The core- and pan-genome exhibited comparable distribution of functions to those of the individual strains. In contrast, the total functional distribution of singletons varied significantly. Similar patterns between strains, the core genome and the pan-genome were also observed in COG functional categories, while singletons again showed distinct profile (Figure 6B). Overall, both KEGG and COG annotations indicated that the main metabolic pathways and cellular processes are largely conserved across all four *L. monocytogenes* strains, while the presence of singletons likely reflects strain-specific adaptations to distinct environmental niches.

### 3.6. Virulence Potential

A total of 40 virulence-associated genes were identified in the *L. monocytogenes* strains (Table 3). Certain genes were detected across all four genomes, including key pathogenicity determinants such as *prfA*, *plcA*, *plcB*, *hly*, *actA*, and *mpl*, which constitute the *L. monocytogenes* pathogenicity island 1 (LIPI-1) essential for intracellular survival and spread within the host [11,12,75,76,77,78]. The genomes also carried multiple internalin genes (*inlA, inlB, inlC, inlF,* and *inlK*) mediating adhesion, invasion, and immune evasion [12,79], together with the surface adhesin vip gene whose product interacts with host Gp96 and promotes epithelial internalization [80,81]. In addition, the *lntA* gene, encoding a nuclear-targeted protein that modulates host immune responses through interference with the BAHD1 chromatin repressor [82], was also present in all strains. Further virulence factors involved in host interaction and colonization, including *oatA*, *bsh*, *prsA2*, *iap, lap, lapB,* and *fbpA* [83,84,85,86] were found to be present.

Strains A2D9 and EGD-e both carried the additional internalin gene *inlJ*, which promotes bacterial adherence [87]. Strain A2D9 also harbored *pdgA,* involved in immune evasion [88,89], as well as the *aut* and *ami* genes, which contribute to bacterial adhesion, invasion and binding to host cells [90,91]. The presence of these genes suggests an extended repertoire of surface- and cell-wall–associated pathogenicity traits, potentially enhancing the capacity of A2D9 to adhere, invade, and persist within the host.

The genome of A2D10 also harbored the complete *lls* gene cluster (*llsA*, *llsB*, *llsD, llsG, llsH*, *llsP*, *llsX,* and *llsY*), forming the *L. monocytogenes* pathogenicity island 3 (LIPI-3), which encodes the thiazole/oxazole–modified microcin listeriolysin S (LLS) [77,92,93]. This peptide, produced by hypervirulent *L. monocytogenes* strains, has been associated with severe listeriosis outbreaks and enables modification of the host gut microbiota, promoting efficient intestinal colonization and invasion of internal organs [93]. These findings are consistent with the fatal clinical outcome of this strain. Strain C5 did not harbor any unique virulence genes compared to other strains.

### 3.7. Antimicrobial Resistance

In vitro antimicrobial testing of all *L. monocytogenes* strains revealed identical susceptibility profiles. All isolates were resistant to cefoxitin, oxacillin, daptomycin, and moxifloxacin, exhibited intermediate susceptibility to ciprofloxacin and remained sensitive to ampicillin, benzylpenicillin, gentamicin, erythromycin, clindamycin, quinupristin/dalfopristin, linezolid, teicoplanin, vancomycin, tigecycline, and trimethoprim/sulfamethoxazole. Resistance to cefoxitin and oxacillin is recognized as intrinsic in *L. monocytogenes*, reflecting the low affinity of its penicillin-binding proteins for cephalosporins and certain β-lactams antibiotics [13]. Reduced susceptibility to daptomycin has also been reported in *L. monocytogenes* [94]. Similarly, intermediate or reduced responses to fluoroquinolones such as ciprofloxacin and moxifloxacin are consistent with previous observations [95,96], while the overall susceptibility to ampicillin, benzylpenicillin, gentamicin, erythromycin, clindamycin, linezolid, and vancomycin reflects the typical antimicrobial profile of this species [13,97].

Genome screening revealed the presence of intrinsic AMR genes *fosX*, *norB*, *mprF*, and *lin* (Table 4). These genes confer resistance to fosfomycin, fluoroquinolones, cationic antimicrobial peptides, and lincosamides, respectively [14,98]. The presence of *fosX*, *norB*, and *mprF* is consistent with the observed resistance to β-lactams, daptomycin, and fluoroquinolones [14,99].

Furthermore, all strains carried the *mdrL* gene, which encodes a multidrug efflux pump responsible for the export of macrolides, cefotaxime, heavy metals, ethidium bromide and benzalkonium chloride [13]. The combined presence of *mdrL*, *fosX*, *norB*, *mprF*, and *lin* highlights the conserved multidrug resistance profile of *L. monocytogenes* strains supporting the findings of our in vitro antimicrobial tests [13,100,101].

### 3.8. Defense Mechanisms and Miscellaneous Traits

A variety of defense mechanisms were identified across all *L. monocytogenes* strains (Table 5). Strain C5 possessed type I, II and IIG R–M systems, reflecting an enhanced ability to recognize and degrade invading foreign DNA [102,103,104]. Strain A2D9 carried a CRISPR–Cas Type IB system together with an Iet system composed of the ATPase and protease components [105], as well as a retron I-C defense module [50,106], suggesting an expanded defense repertoire. A type II R-M system was also predicted for strain A2D10 alongside an RloC protein, shown to function through tRNA cleavage [107]. Additionally, this strain harbored abortive infection system (Abi) [108,109] and defense-associated reverse transcriptase (DRT) systems, which are known to restrict phage infection and inhibit protein translation [109,110]. Finally, the R-M type II and Abi defense systems were also predicted for EGD-e strain.

All strains contained one questionable and one incomplete prophage region, except for EGD-e, which harbored two questionable regions. Additionally, strain C5 contained one intact prophage region. Furthermore, strains C5, A2D9 and EGD-e each carried four genomic islands, while strain A2D10 harbored two. The observed differences in defense systems, prophage regions, and genomic islands highlight the genomic plasticity of the *L. monocytogenes* strains and their ongoing adaptation through horizontal gene transfer. It should be noted that all findings of this study are based on bioinformatics annotation and have not undergone any experimental functional validation.

## 4. Conclusions

The comparative genomic analysis of three novel *L. monocytogenes* strains provided comprehensive insights into their genetic composition, functional potential, virulence traits, antimicrobial resistance, defense mechanisms and genomic plasticity. All genomes exhibited high completeness and a large core-genome, while the presence of unique genes highlighted genomic diversity. MLST analysis confirmed that the strains represented distinct sequence types. Functional annotation revealed that most genes were involved in fundamental metabolic processes, while the distribution of singletons suggested strain-specific functions that may contribute to environmental persistence or host adaptation.

Variation in virulence genes suggested strain-specific differences in pathogenic potential, most notably the presence of the LIPI-3 island in A2D10, which is associated with enhanced virulence and may help explain the severe clinical outcome linked to this strain. The conserved resistance and susceptibility patterns across all genomes indicated the typical antimicrobial profile of *L. monocytogenes*, supporting the use of ampicillin or benzylpenicillin as first-line therapy in clinical practice. The identification of resistance determinants such as *fosX*, *norB*, *mprF* and *lin* further confirms the current reliability of first-line therapies but underscores the necessity of ongoing genomic surveillance to detect any future emergence of high-risk acquired traits. Furthermore, the presence of multiple bacterial defense systems, prophage regions and genomic islands suggests mechanisms that may facilitate adaptation to diverse ecological niches, including food-processing environments and the human host.

The identified genomic features reveal key traits relevant both to clinical management strategies and the survival of *L. monocytogenes* in food-processing environments. Further investigation of these strains is warranted to elucidate the functional significance of the identified genomic features and their contribution to *L. monocytogenes* virulence and environmental adaptation.

## Figures and Tables

**Table 1:** Characteristics of genome assemblies of *L. monocytogenes* strains according to CheckM2.

Strain	Completeness (%)	Contamination (%)	GC Content (%)	Size (bp)
C5	99.99	0.61	38	2,985,720
A2D9	99.99	0.81	38	2,873,619
A2D10	99.98	0.69	38	2,902,195

**Table 2:** Key genomic features of *L. monocytogenes* strains.

Strain	Number of Genes	Number of CDS	Number of tRNAs Genes	Number of rRNAs
C5	2971	2903	67	18
A2D9	2837	2769	67	18
A2D10	2870	2802	67	18

**Table 3:** Virulence genes identified in the *L. monocytogenes* strains.

Gene	Function	C5	A2D9	A2D10	EGD-e
*prfA*	Listeriolysin positive regulatory protein	+	+	+	+
*plcA*	Phosphatidylinositol-specific phospholipase c	+	+	+	+
*plcB*	Phospholipase C	+	+	+	+
*hly*	Listeriolysin O precursor	+	+	+	+
*mpl*	Zinc metalloproteinase precursor	+	+	+	+
*actA*	Actin-assembly inducing protein precursor	+	+	+	+
*clpC*	Endopeptidase Clp ATP-binding chain C	+	+	+	+
*vip*	surface aghesin Vip	+	+	+	+
*inlA*	Internalin A	+	+	+	+
*inlB*	Internalin B	+	+	+	+
*inlC*	Internalin C	+	+	+	+
*inlF*	Internalin F	+	+	+	+
*inlJ*	Internalin J	-	+	-	+
*inlK*	Internalin K	+	+	+	+
*pdgA*	Peptidoglycan *N*-deacetylase	-	+	-	+
*lntA*	Listeria nuclear targeted protein A	+	+	+	+
*iap/cwhA*	P60 extracellular protein invasion associated protein Iap	+	+	+	+
*hpt*	Hexose phosphate transport protein	+	+	+	+
*iplA1*	Lipoate protein ligase	+	+	+	+
*clpE*	ATP-dependent protease	+	+	+	+
*aut*	Autolysin	-	+	-	+
*llsA*	Listeriolysin S	-	-	+	-
*llsB*	SagB family dehydrogenase LlsB	-	-	+	-
*llsD*	Streptolysin-assossiated protein LlsD	-	-	+	-
*llsG*	ABC transporter ATP-binding protein LlsG	-	-	+	-
*llsH*	ABC transporter permease protein LlsH	-	-	+	-
*llsP*	CAAX amino terminal protease LlsP	-	-	+	-
*llsX*	Protein LlsX	-	-	+	-
*llsY*	Protein LlsY	-	-	+	-
*oatA*	Peptidoglycan O-acetyltransferase	+	+	+	+
*lap*	Listeria adhesion protein	+	+	+	+
*lapB*	Listeria adhesion protein LapB	+	+	+	+
*fbpA*	Fibronectin-binding protein	+	+	+	+
*lspA*	Signal peptidase II	+	+	+	+
*lpeA*	Lipoprotein promoting cell invasion	+	+	+	+
*bsh*	Bile salt hydrolase	+	+	+	+
*prsA2*	Post translocation chaperone PrsA2	+	+	+	+
*clpP*	ATP-dependent Clp protease proteolytic subunit	+	+	+	+
*gtcA*	Wall teichoic acid glycosylation protein GtcA	+	+	+	+
*ami*	Autolysin amidase adhesin	-	+	-	+

**Table 4:** Antimicrobial resistance genes present in *L. monocytogenes* strains.

Gene	Function	Resistance	C5	A2D9	A2D10	EGD-e
*lin*	Lin protein	Lincosamides	+	+	+	+
*fosX*	FosX enzyme	Fosfomycin	+	+	+	+
*norB*	NorB multidrug efflux pump	Fluoroquinolones	+	+	+	+
*mprF*	MprF integral membrane protein	Cationic peptides	+	+	+	+
*mdrL*	MdrL efflux pump	Multidrug resistance	+	+	+	+

**Table 5:** Summary of defense systems and horizontally acquired elements identified in *L. monocytogenes* strains.

Strain	Defense Systems	Prophage Regions	Genomic Islands
C5	R-M type I, R-M type II, R-M type IIG	3	4
A2D9	CRISPR-Cas type-IB, IetAS, retron I-C	2	4
A2D10	R-M type II, RloC, DRT2, Abi2	2	2
EGD-e	R-M type II, AbiH	2	4

## Data Availability

The whole-genome shotgun projects have been deposited in GenBank under the Bioproject IDs PRJNA1173118 and PRJNA1375526.

## References

[1] Disson O., Charlier C., Pérot P., Leclercq A., Paz R.N., Kathariou S., Tsai Y.-H., Lecuit M. (2025). Listeriosis. Nat. Rev. Dis. Primers.

[2] Moura A., Criscuolo A., Pouseele H., Maury M.M., Leclercq A., Tarr C., Björkman J.T., Dallman T., Reimer A., Enouf V. (2016). Whole genome-based population biology and epidemiological surveillance of *Listeria monocytogenes*. Nat. Microbiol..

[3] Radoshevich L., Cossart P. (2018). *Listeria monocytogenes*: Towards a complete picture of its physiology and pathogenesis. Nat. Rev. Microbiol..

[4] Swaminathan B., Gerner-Smidt P. (2007). The epidemiology of human listeriosis. Microbes Infect..

[5] Kuenne C., Billion A., Mraheil M.A., Strittmatter A., Daniel R., Goesmann A., Barbuddhe S., Hain T., Chakraborty T. (2013). Reassessment of the *Listeria monocytogenes* pan-genome reveals dynamic integration hotspots and mobile genetic elements as major components of the accessory genome. BMC Genom..

[6] Lachtara B., Wieczorek K., Osek J. (2022). Genetic diversity and relationships of *Listeria monocytogenes* serogroup IIa isolated in Poland. Microorganisms.

[7] Liu D., Lawrence M.L., Wiedmann M., Gorski L., Mandrell R.E., Ainsworth A.J., Austin F.W. (2006). *Listeria monocytogenes* subgroups IIIA, IIIB, and IIIC delineate genetically distinct populations with varied pathogenic potential. J. Clin. Microbiol..

[8] Orsi R.H., den Bakker H.C., Wiedmann M. (2011). *Listeria monocytogenes* lineages: Genomics, evolution, ecology, and phenotypic characteristics. Int. J. Med. Microbiol..

[9] Zamudio R., Haigh R.D., Ralph J.D., De Ste Croix M., Tasara T., Zurfluh K., Kwun M.J., Millard A.D., Bentley S.D., Croucher N.J. (2020). Lineage-specific evolution and gene flow in *Listeria monocytogenes* are independent of bacteriophages. Environ. Microbiol..

[10] Orsi R.H., Wiedmann M. (2016). Characteristics and distribution of *Listeria* spp., including *Listeria* species newly described since 2009. Appl. Microbiol. Biotechnol..

[11] De Las Heras A., Cain R.J., Bielecka M.K., Vázquez-Boland J.A. (2011). Regulation of *Listeria* virulence: PrfA master and commander. Curr. Opin. Microbiol..

[12] Lecuit M. (2020). *Listeria monocytogenes*, a model in infection biology. Cell Microbiol..

[13] Luque-Sastre L., Arroyo C., Fox E.M., McMahon B.J., Bai L., Li F., Fanning S. (2018). Antimicrobial resistance in *Listeria* species. Microbiol. Spectr..

[14] Scortti M., Han L., Alvarez S., Leclercq A., Moura A., Lecuit M., Vazquez-Boland J. (2018). Epistatic control of intrinsic resistance by virulence genes in *Listeria*. PLoS Genet..

[15] Kim J.W., Kathariou S. (2009). Temperature-dependent phage resistance of *Listeria monocytogenes* epidemic clone II. Appl. Environ. Microbiol..

[16] Osuna B.A., Karambelkar S., Mahendra C., Christie K.A., Garcia B., Davidson A.R., Kleinstiver B.P., Kilcher S., Bondy-Denomy J. (2020). *Listeria* phages induce Cas9 degradation to protect lysogenic genomes. Cell Host Microbe.

[17] Xu X., Gu P. (2024). Overview of phage defense systems in bacteria and their applications. Mol. Sci..

[18] Georjon H., Bernheim A. (2023). The highly diverse antiphage defence systems of bacteria. Nat. Rev. Microbiol..

[19] Bobay L.M., Ochman H. (2017). The evolution of bacterial genome architecture. Front. Genet..

[20] Gkerekou M.A., Kaparakou E.H., Tarantilis P.A., Skandamis P.N. (2023). Studying the metabolic factors that may impact the growth of co-cultured *Listeria monocytogenes* strains at low temperature. Food Res. Int..

[21] EUCAST (2024). European Committee on Antimicrobial Susceptibility Testing Disk Diffusion Test Methodology. https://www.eucast.org/ast_of_bacteria/disk_diffusion_methodology.

[22] Mölder F., Jablonski K.P., Letcher B., Hall M.B., van Dyken P.C., Tomkins-Tinch C.H., Sochat V., Forster J., Vieira F.G., Meesters C. (2021). Sustainable data analysis with Snakemake. F1000Research.

[23] De Coster W., Rademakers R. (2023). NanoPack2: Population-scale evaluation of long-read sequencing data. Bioinformatics.

[24] Wick R.R., Judd L.M., Gorrie C.L., Holt K.E. (2017). Completing bacterial genome assemblies with multiplex MinION sequencing. Microb. Genom..

[25] Andrews S. (2010). FastQC: A Quality Control Tool for High Throughput Sequence Data. https://www.bioinformatics.babraham.ac.uk/projects/fastqc.

[26] Kolmogorov M., Yuan J., Lin Y., Pevzner P.A. (2019). Assembly of long, error-prone reads using repeat graphs. Nat. Biotechnol..

[27] Lee J.Y., Kong M., Oh J., Lim J., Chung S.H., Kim J.M., Kim J.-S., Kim K.-H., Yoo J.-C., Kwak W. (2021). Comparative evaluation of Nanopore polishing tools for microbial genome assembly and polishing strategies for downstream analysis. Sci. Rep..

[28] Li H., Durbin R. (2010). Fast and accurate long-read alignment with Burrows-Wheeler transform. Bioinformatics.

[29] Walker B.J., Abeel T., Shea T., Priest M., Abouelliel A., Sakthikumar S., Cuomo C.A., Zeng Q., Wortman J., Young S.K. (2014). Pilon: An integrated tool for comprehensive microbial variant detection and genome assembly improvement. PLoS ONE.

[30] Hunt M., Silva N.D., Otto T.D., Parkhill J., Keane J.A., Harris S.R. (2015). Circlator: Automated circularization of genome assemblies using long sequencing reads. Genome Biol..

[31] Pellow D., Mizrahi I., Shamir R. (2020). PlasClass improves plasmid sequence classification. PLoS Comput. Biol..

[32] Chaumeil P.A., Mussig A.J., Hugenholtz P., Parks D.H. (2022). GTDB-Tk v2: Memory friendly classification with the genome taxonomy database. Bioinformatics.

[33] Seemann T. (2014). Prokka: Rapid prokaryotic genome annotation. Bioinformatics.

[34] Manni M., Berkeley M.R., Seppey M., Simão F.A., Zdobnov E.M. (2021). BUSCO update: Novel and streamlined workflows along with broader and deeper phylogenetic coverage for scoring of eukaryotic, prokaryotic, and viral genomes. Mol. Biol. Evol..

[35] Chklovski A., Parks D.H., Woodcroft B.J., Tyson G.W. (2023). CheckM2: A rapid, scalable and accurate tool for assessing microbial genome quality using machine learning. Nat. Methods.

[36] Meier-Kolthoff J.P., Goker M. (2019). TYGS is an automated high-throughput platform for state-of-the-art genome-based taxonomy. Nat. Commun..

[37] Darling A.C.E., Mau B., Blattner F.R., Perna N.T. (2004). Mauve: Multiple alignment of conserved genomic sequence with rearrangements. Genome Res..

[38] Darzentas N. (2010). Circoletto: Visualizing sequence similarity with Circos. Bioinformatics.

[39] Edgar R.C. (2004). MUSCLE: Multiple sequence alignment with high accuracy and high throughput. Nucleic Acids Res..

[40] Felsenstein J. (2018). PHYLIP—Phylogeny Inference Package. https://phylipweb.github.io/phylip.

[41] Richter M., Rosselló-Móra R., Oliver Glöckner F., Peplies J. (2015). JSpeciesWS: A web server for prokaryotic species circumscription based on pairwise genome comparison. Bioinformatics.

[42] Pasteur BIGSdb (2024). BIGSdb. http://bigsdb.pasteur.fr/news/.

[43] Kanehisa M., Sato Y., Morishima K. (2016). BlastKOALA and GhostKOALA: KEGG tools for functional characterization of genome and metagenome sequences. J. Mol. Biol..

[44] Cantalapiedra C.P., Hernández-Plaza A., Letunic I., Bork P., Huerta-Cepas J. (2021). ΕggNOG-mapper v2: Functional annotation, orthology assignments, and domain prediction at the metagenomic scale. Mol. Biol. Evol..

[45] Seemann T. (2014). Abricate. https://github.com/tseemann/abricate.

[46] Gupta S.K., Padmanabhan B.R., Diene S.M., Lopez-Rojas R., Kempf M., Landraud L., Rolain J.M. (2014). ARG-ANNOT, a new bioinformatic tool to discover antibiotic resistance genes in bacterial genomes. Antimicrob. Agents Chemother..

[47] Jia B., Raphenya A.R., Alcock B., Waglechner N., Guo P., Tsang K.K., Lago B.A., Dave B.M., Pereira S., Sharma A.N. (2017). CARD 2017: Expansion and model-centric curation of the comprehensive antibiotic resistance database. Nucleic Acids Res..

[48] Doster E., Lakin S.M., Dean C.J., Wolfe C., Young J.G., Boucher C., Belk K.E., Noyes N.R., Morley P.S. (2019). MEGARes 2.0: A database for classification of antimicrobial drug, biocide and metal resistance determinants in metagenomic sequence data. Nucleic Acids Res..

[49] Liu B., Zheng D., Zhou S., Chen L., Yang J. (2022). VFDB 2022: A general classification scheme for bacterial virulence factors. Nucleic Acids Res..

[50] Tesson F., Hervé A., Mordret E., Touchon M., d’Humières C., Cury J., Bernheim A. (2022). Systematic and quantitative view of the antiviral arsenal of prokaryotes. Nat. Commun..

[51] Arndt D., Grant J.R., Marcu A., Sajed T., Pon A., Liang Y., Wishart D.S. (2016). PHASTER: A better, faster version of the PHAST phage search tool. Nucleic Acids Res..

[52] Bertelli C., Laird M.R., Williams K.P., Lau B.Y., Hoad G., Winsor G.L., Brinkman F.S.L. (2017). IslandViewer 4: Expanded prediction of genomic islands for larger-scale datasets. Nucleic Acids Res..

[53] Qi Y., Cao Q., Zhao X., Tian C., Li T., Shi W., Wei H., Song C., Xue H., Gou H. (2024). Comparative genomic analysis of pathogenic factors of *Listeria* spp. using whole-genome sequencing. BMC Genom..

[54] Lachtara B., Osek J., Wieczorek K. (2021). Molecular typing of Listeria monocytogenes IVb serogroup isolated from food and food production environments in Poland. Pathogens.

[55] Briers Y., Klumpp J., Schuppler M., Loessner M.J. (2011). Genome sequence of *Listeria monocytogenes* Scott A, a clinical isolate from a food-borne listeriosis outbreak. J. Bacteriol..

[56] Liu D. (2006). Identification, subtyping and virulence determination of *Listeria monocytogenes*, an important foodborne pathogen. J. Med. Microbiol..

[57] Maijala R., Lyytikäinen O., Johansson T., Autio T., Aalto T., Haavisto L., Honkanen-Buzalski T. (2001). Exposure of *Listeria monocytogenes* within an epidemic caused by butter in Finland. Int. J. Food Microbiol..

[58] Rychli K., Müller A., Zaiser A., Schoder D., Allerberger F., Wagner M., Schmitz-Esser S. (2014). Genome sequencing of *Listeria monocytogenes* “Quargel” listeriosis outbreak strains reveals two different strains with distinct in vitro virulence potential. PLoS ONE.

[59] Nelson K.E., Fouts D.E., Mongodin E.F., Ravel J., DeBoy R.T., Kolonay J.F., Rasko D.A., Angiuoli S.V., Gill S.R., Paulsen I.T. (2004). Whole genome comparisons of serotype 4b and 1/2a strains of the food-borne pathogen *Listeria monocytogenes* reveal new insights into the core genome components of this species. Nucleic Acids Res..

[60] Tan W., Wang G., Pan Z., Yin Y., Jiao X. (2015). Complete genome sequence of *Listeria monocytogenes* NTSN, a serovar 4b and animal source strain. Genome Announc..

[61] Kichemazova N., Zaytsev S., Saltykov Y., Larionova O., Zaberezhny A., Feodorova V. (2025). MLST evidence of two different sequence types of *Listeria monocytogenes* strains used for commercial veterinary listeriosis vaccines. Zoonoses.

[62] Salcedo C., Arreaza L., Alcala B., de la Fuente L., Vazquez J.A. (2003). Development of a multilocus sequence typing method for analysis of *Listeria monocytogenes* clones. J. Clin. Microbiol..

[63] Mafuna T., Matle I., Magwedere K., Pierneef R.E., Reva O.N. (2021). Whole genome-based characterization of *Listeria monocytogenes* isolates recovered from the food chain in South Africa. Front. Microbiol..

[64] Nielsen E.M., Björkman J.T., Kiil K., Grant K., Dallman T., Painset A., Amar C., Roussel S., Guillier L., Félix B. (2017). Closing gaps for performing a risk assessment on *Listeria monocytogenes* in ready-to-eat (RTE) foods: Activity 3, the comparison of isolates from different compartments along the food chain, and from humans using whole genome sequencing (WGS) analysis. EFSA Support. Publ..

[65] Rychli K., Stessl B., Szakmary-Brändle K., Strauß A., Wagner M., Schoder D. (2018). *Listeria monocytogenes* isolated from illegally imported food products into the European Union harbor different virulence factor variants. Genes.

[66] Toledo V., den Bakker H.C., Hormazábal J.C., González-Rocha G., Bello-Toledo H., Toro M., Moreno-Switt A.I. (2018). Genomic diversity of *Listeria monocytogenes* isolated from clinical and non-clinical samples in Chile. Genes.

[67] Cabal A., Pietzka A., Huhulescu S., Allerberger F., Ruppitsch W., Schmid D. (2019). Isolate-based surveillance of *Listeria monocytogenes* by whole genome sequencing in Austria. Front. Microbiol..

[68] Wagner E., Zaiser A., Leitner R., Quijada N.M., Pracser N., Pietzka A., Ruppitsch W., Schmitz-Esser S., Wagner M., Rychli K. (2020). Virulence characterization and comparative genomics of *Listeria monocytogenes* sequence type 155 strains. BMC Genom..

[69] Cheng Y., Dong Q., Liu Y., Liu H., Zhang H., Wang X. (2022). Systematic review of *Listeria monocytogenes* from food and clinical samples in Chinese mainland from 2010 to 2019. Food Qual. Saf..

[70] Dreyer M., Aguilar-Bultet L., Rupp S., Guldimann C., Stephan R., Schock A., Otter A., Schupbach G., Brisse S., Lecuit M. (2016). *Listeria monocytogenes* sequence type 1 is predominant in ruminant rhombencephalitis. Sci. Rep..

[71] Silva A., Silva V., Gomes J.P., Coelho A., Batista R., Saraiva C., Esteves A., Martins Â., Contente D., Diaz-Formoso L. (2024). *Listeria monocytogenes* from food products and food associated environments: Antimicrobial resistance, genetic clustering and biofilm insights. Antibiotics.

[72] Wieczorek K., Bomba A., Osek J. (2020). Whole-genome sequencing-based characterization of *Listeria monocytogenes* from fish and fish production environments in Poland. Mol. Sci..

[73] Ragon M., Wirth T., Hollandt F., Lavenir R., Lecuit M., Le Monnier A., Brisse S. (2008). A new perspective on *Listeria monocytogenes* evolution. PLoS Pathog..

[74] Li H., Wang P., Lan R., Luo L., Cao X., Wang Y., Wang Y., Li H., Zhang L., Ji S. (2018). Risk factors and level of *Listeria monocytogenes* contamination of raw pork in retail markets in China. Front. Microbiol..

[75] Glomski I.J., Gedde M.M., Tsang A.W., Swanson J.A., Portnoy D.A. (2002). The *Listeria monocytogenes* hemolysin has an acidic pH optimum to compartmentalize activity and prevent damage to infected host cells. J. Cell Biol..

[76] Mitchell G., Ge L., Huang Q., Chen C., Kianian S., Roberts M.F., Schekman R., Portnoy D.A. (2015). Avoidance of autophagy mediated by PlcA or ActA is required for *Listeria monocytogenes* growth in macrophages. Infect. Immun..

[77] Song Y., Gao B., Cai H., Qin X., Xia X., Dong Q., Hirata T., Li Z. (2025). Comparative analysis of virulence in *Listeria monocytogenes*: Insights from genomic variations and in vitro cell-based studies. Int. J. Food Microbiol..

[78] Wiktorczyk-Kapischke N., Skowron K., Wałecka-Zacharska E. (2023). Genomic and pathogenicity islands of *Listeria monocytogenes*—Overview of selected aspects. Front. Mol. Biosci..

[79] Pedroni L., Ghidini S., Vazquez J., Luque F.J., Dellafiora L. (2025). Modeling the anti-adhesive role of punicalagin against *Listeria monocytogenes* from the analysis of the interaction between internalin a and e-cadherin. Int. J. Mol. Sci..

[80] Cabanes D., Sousa S., Cebriá A., Lecuit M., García-del Portillo F., Cossart P. (2005). Gp96 is a receptor for a novel *Listeria monocytogenes* virulence factor, Vip, a surface protein. Embo J..

[81] Camejo A., Carvalho F., Reis O., Leitao E., Sousa S., Cabanes D. (2011). The arsenal of virulence factors deployed by *Listeria monocytogenes* to promote its cell infection cycle. Virulence.

[82] Lebreton A., Lakisic G., Job V., Fritsch L., Tham T.N., Camejo A., Matteï P.-J., Regnault B., Nahori M.-A., Cabanes D. (2011). A Bacterial protein targets the BAHD1 chromatin complex to stimulate type III interferon response. Science.

[83] Agbavor C., Zimnicka A., Kumar A., George J.L., Torres M., Prehna G., Alonzo F., Durrant J.D., Freitag N.E., Cahoon L.A. (2024). The chaperone PrsA2 regulates the secretion, stability, and folding of listeriolysin O during *Listeria monocytogenes* infection. mBio.

[84] Burkholder K.M., Bhunia A.K. (2010). *Listeria monocytogenes* uses *Listeria* adhesion protein (LAP) to promote bacterial transepithelial translocation and induces expression of LAP receptor Hsp60. Infect. Immun..

[85] Ingeborg H., Klein D.K., Lehner A., Bubert A., Branda Ernst B., Wagner M. (2001). Detection and quantification of the iap gene of *Listeria monocytogenes* and *Listeria innocua* by a new real-time quantitative PCR assay. Res. Microbiol..

[86] Quereda J.J., Morón-García A., Palacios-Gorba C., Dessaux C., García-Del Portillo F., Pucciarelli M.G., Ortega A.D. (2021). Pathogenicity and virulence of *Listeria monocytogenes*: A trip from environmental to medical microbiology. Virulence.

[87] Sabet C., Toledo-Arana A., Personnic N., Lecuit M., Dubrac S., Poupel O., Gouin E., Nahori M.A., Cossart P., Bierne H. (2008). The *Listeria monocytogenes* virulence factor InlJ is specifically expressed in vivo and behaves as an adhesin. Infect. Immun..

[88] Parra-Flores J., Daza-Prieto B., Chavarria P., Troncoso M., Stoger A., Figueroa G., Mancilla-Rojano J., Cruz-Cordova A., Martinovic A., Ruppitsch W. (2025). From traditional typing to genomic precision: Whole-genome sequencing of *Listeria monocytogenes* isolated from refrigerated foods in Chile. Foods.

[89] Pyz-Lukasik R., Paszkiewicz W., Kielbus M., Ziomek M., Gondek M., Domaradzki P., Michalak K., Pietras-Ozga D. (2022). Genetic diversity and potential virulence of *Listeria monocytogenes* isolates originating from polish artisanal cheeses. Foods.

[90] Fern Tan M., Siow C.C., Dutta A., Mutha N.V., Wee W.Y., Heydari H., Tan S.Y., Ang M.Y., Wong G.J., Choo S.W. (2015). Development of ListeriaBase and comparative analysis of *Listeria monocytogenes*. BMC Genom..

[91] Milohanic E., Jonquières R., Cossart P., Berche P., Gaillard J. (2001). The autolysin Ami contributes to the adhesion of *Listeria monocytogenes* to eukaryotic cells via its cell wall anchor. Mol. Microbiol..

[92] Lee S. (2020). Bacteriocins of *Listeria monocytogenes* and their potential as a virulence factor. Toxins.

[93] Meza-Torres J., Lelek M., Quereda J.J., Sachse M., Manina G., Ershov D., Tinevez J.Y., Radoshevich L., Maudet C., Chaze T. (2021). Listeriolysin S: A bacteriocin from *Listeria monocytogenes* that induces membrane permeabilization in a contact-dependent manner. Proc. Natl. Acad. Sci. USA.

[94] Spanjaard L., Vandenbroucke-Grauls C.M. (2008). Activity of daptomycin against *Listeria monocytogenes* isolates from cerebrospinal fluid. Antimicrob. Agents Chemother..

[95] Charpentier E., Courvalin P. (1999). Antibiotic resistance in *Listeria* spp.. Antimicrob. Agents Chemother..

[96] Grayo S., Join-Lambert O., Desroches M.C., Le Monnier A. (2008). Comparison of the in vitro efficacies of moxifloxacin and amoxicillin against *Listeria monocytogenes*. Antimicrob. Agents Chemother..

[97] Conter M., Paludi D., Zanardi E., Ghidini S., Vergara A., Ianieri A. (2009). Characterization of antimicrobial resistance of foodborne *Listeria monocytogenes*. Int. J. Food Microbiol..

[98] Manqele A., Adesiyun A., Mafuna T., Pierneef R., Moerane R., Gcebe N. (2024). Virulence potential and antimicrobial resistance of *Listeria monocytogenes* isolates obtained from beef and beef-based products deciphered using whole-genome sequencing. Microorganisms.

[99] Ernst C.M., Peschel A. (2019). MprF-mediated daptomycin resistance. Int. J. Med. Microbiol..

[100] Krawczyk-Balska A., Markiewicz Z. (2016). The intrinsic cephalosporin resistome of *Listeria monocytogenes* in the context of stress response, gene regulation, pathogenesis and therapeutics. J. Appl. Microbiol..

[101] Lungu B., Ricke S.C., Johnson M.G. (2009). Growth, survival, proliferation and pathogenesis of *Listeria monocytogenes* under low oxygen or anaerobic conditions: A review. Anaerobe.

[102] Oliveira P.H., Touchon M., Rocha E.P.C. (2014). The interplay of restriction-modification systems with mobile genetic elements and their prokaryotic hosts. Nucleic Acids Res..

[103] Shen B.W., Xu D., Chan S.H., Zheng Y., Zhu Z., Xu S.Y., Stoddard B.L. (2011). Characterization and crystal structure of the type IIG restriction endonuclease RM.BpuSI. Nucleic Acids Res..

[104] Tock M.R., Dryden D.T.F. (2005). The biology of restriction and anti-restriction. Curr. Opin. Microbiol..

[105] Gao L., Altae-Tran H., Böhning F., Makarova K.S., Segel M., Schmid-Burgk J.L., Koob J., Wolf Y.I., Koonin E.V., Zhang F. (2020). Diverse enzymatic activities mediate antiviral immunity in prokaryotes. Science.

[106] Millman A., Bernheim A., Stokar-Avihail A., Fedorenko T., Voichek M., Leavitt A., Oppenheimer-Shaanan Y., Sorek R. (2020). Bacterial retrons function in anti-phage defense. Cell.

[107] Davidov E., Kaufmann G. (2008). RloC: A wobble nucleotide-excising and zinc-responsive bacterial tRNase. Mol. Microbiol..

[108] Aframian N., Eldar A. (2023). Abortive infection antiphage defense systems: Separating mechanism and phenotype. Trends Microbiol..

[109] Mestre M.R., Gao L.A., Shah S.A., López-Beltrán A., González-Delgado A., Martínez-Abarca F., Iranzo J., Redrejo-Rodríguez M., Zhang F., Toro N. (2022). UG/Abi: A highly diverse family of prokaryotic reverse transcriptases associated with defense functions. Nucleic Acids Res..

[110] Dy R.L., Przybilski R., Semeijn K., Salmond G.P., Fineran P.C. (2014). A widespread bacteriophage abortive infection system functions through a Type IV toxin-antitoxin mechanism. Nucleic Acids Res..

